# Labour roots and migration routes: precarious employment as driver of irregular migration amongst women workers

**DOI:** 10.3389/fsoc.2025.1636127

**Published:** 2025-08-12

**Authors:** Rosa Lázaro Castellanos

**Affiliations:** Department of Social Anthropology, Faculty of Geography and History, University of Barcelona, Barcelona, Spain

**Keywords:** migrant women, precarious employment, social exclusion, psychosocial impact, health and housing, marginalization

## Abstract

In Spain, the number of non-EU foreigners in an irregular administrative situation is relatively low, as priority is given to ensuring that legal residency status is the norm. However, individuals with regularized status are always at risk of falling into irregularity, as both residence and employment permits are tied to a labor contract. Through qualitative research, a group of eight women was interviewed to examine inequalities in access to employment, housing, and social security during the pandemic. The findings show that women without formal employment are vulnerable to losing their legal status, becoming homeless, or experiencing stress due to non-compliance with immigration laws. Additionally, the article presents experiences of self-managed support networks created to address both material and psychosocial challenges during the pandemic.

## Introduction

The 1990s were characterised by an upsurge in migration on a global scale, a product of persistent political, social and economic inequality between regions and countries. Three characteristics stood out in the migrations of the 1990s: the feminisation of flows, child migration, migrations resulting from climate change (Ortega, 2015; INFORME ES 08, 2021), and unemployment is derived from neoliberal policies that have made life precarious, and expelled people from their communities.

In the European Union, Spain and France are the largest recipients of non-EU immigrants. Spain is characterised by the fact that the migratory flows it receives are mainly women from the global South, or with historical links such as colonialism. The ageing of the European population, the emerging care economy, socio-cultural ties, and expectations of better life opportunities are sufficient reasons for women to come to Spain (Pedone et al., 2012).

Authors such as Pedone et al. (2012), Riopedre (2016) and Salas (2019) argue that Latin American women who have arrived in Spain stand out for carrying out their own migration projects, for being the initiators of family reunification processes for children and men, and for being responsible for family subsistence both in the country of origin and at destination. The mobility of these women is generally associated with employment in low-skilled, temporary, precarious or irregular sectors, such as care work. Once in Spain, some have achieved permanent regularization and others became irregular by overstaying, working without a permit or with irregular residence (Pedone et al., 2012). In general, the EU does not have exact figures on irregular entrants; the records it has are of those who have crossed the Mediterranean by sea.

Irregular migration has currently become a topic of social debate and the focus of control policies and laws (Ortega, 2015). With the aim of reducing it, different management mechanisms have been developed, such as increased entry restrictions, the establishment of differences in access to the labour market, the circulation of anti-immigrant discourses (González, 2021), or the detention of people who do not have residence or employment permits. Most of these measures imply the criminalisation of irregular migration.

Although Spain has had some tolerance towards irregular labor immigration (Aja, 2004; González, 2021; Castles, 2010), especially in relation to care work for native families, this tolerance has been inconsistent and limited. In Spain, informal care work is the sector that generates the most employment for migrant women.

The care economy is a path to regularization for immigrant women. However, regularization depends on an employment contract, which is not always easy to obtain. An employment contract enables irregular individuals to benefit from an exceptional immigration law that grants residence permits to those in irregular situations (Salas, 2019). Furthermore, for individuals who have already regularized their status, the employment contract is essential for renewing their residency.

The COVID-19 pandemic in Spain highlighted the importance of immigrant women workers, particularly care workers, but also their vulnerability. Due to the special labor regime, some women were unjustifiably dismissed, others were exposed to contagion, and others were excluded from basic social protection. It should be noted that after the pandemic, Law RD-L 16/2022 eliminated dismissal without just cause, gives access to unemployment benefits, and makes the employment contract more flexible (Molina, 2025).

The criteria of the new law have been simplified, but in practice, the requirements for regularization are difficult to achieve. The contract remains a mechanism for reproducing inequality, as it can become a significant disadvantage for women in a regular and irregular administrative situation. The aim of this paper is to demonstrate, from the experience of migrant women, and in the context of the pandemic, how the state produces the conditions that make people irregular or irregular workers. Irregular status has an impact on the type of access to employment, housing, and mental and physical health.

The article is divided into three main sections. The first shows that migration control has historically been built on criteria of racial exclusion. These criteria are still valid today, but with the addition that the negative narrative on migration, in general, has contributed to its criminalisation. The second section describes the methodology used, as well as the profile of the research participants. A third section presents and analyses the empirical results of the research, highlighting the importance of the figure of labour roots in keeping women in administrative regularity or falling into irregularity. It also describes the impact of the pandemic on employment, health, housing and the strategies developed by women to overcome the crisis. Finally, conclusions are presented.

### Migration management grounded on racial exclusion

Through different eras and socio-economic processes, Western European countries have become recipients of international migration. This has led to their societies being considered diverse and multi-ethnic. This diversity, in terms of migration, has become a concept that affirms socio-economic privileges and exclusions. For example, current migration laws are designed to facilitate access to mobility for Westerners, while people from impoverished regions face extensive restrictions and have few possibilities to migrate legally (Garcia, 2021).

On the other hand, in receiving countries, regular and irregular immigration has become the focus of debate, because the immigrant is accused of altering the social order, of put at risk the Welfare State, and of taking jobs away from native people, which has led to a range of perspectives on migration. Some positions recognise the socio-economic benefits of immigration; other positions argue that there are deep differences with some communities, leading to tensions or outright anti-immigrant and xenophobic rejection. This has allowed governments to use criminal law to criminalise migrant bodies and human mobility.

Historically, anti-immigrant polices have existed. For example, the control policies developed during the nineteenth and twentieth centuries responded to two conditions: on the one hand, economic development and the need for foreign labour; and, on the other hand, to combat diversity in order to unify the community and achieve national identity (Yankelevich, 2015). All countries created legal and bureaucratic institutions to select “ethnically desirable” migrants (Scott and Cook, 2015). For not only foreign labour was in demand, but also people with the capacity to assimilate to the habits and lifestyles of the receiving country.

Exclusion emerges as a practice in nationality and migration control policies. Yankelevich (2015) argues that the regulatory frameworks of international migration are built on criteria of racial exclusion. They emerged at the beginning of the last century, when nobody questioned the idea that there were “superior and inferior races.” For example, in President Roosevelt’s view, the Chinese deserved to be excluded, as they belonged to an inferior race and were incapable of governing themselves in a democracy such as the US (Scott and Cook, 2015). This gave rise to the category of the ‘undesirable’ immigrant, a term that judges certain human groups as a threat to cultural unity.

We do not live in a post-racial world, although selection has apparently ceased to be racial, it is present in discriminatory practices of cultural selection. Current migration control applies health, political, ideological, economic or notions of what constitutes a good immigrant or worker (Scott and Cook, 2015; Garcia, 2021). That is, although the law appears ethnically neutral, there are attempts to classify people into desirable and undesirable, fit and unfit, or assimilable and unassimilable.

Underlying all selection policies is the idea of maintaining white supremacy. This is the reason why states created a legal, and bureaucratic, machinery to select ethnically “desirable” migrants among the millions who migrate (Yankelevich, 2015). For Mbembe (2016), “race” today is actualized under the ideology of security and risk/protection. Race, as a machine producing certain subjects, divides humanity between those who are subjects of the market and debt, and those who are qualified as “superfluous humanity.” For example, the body of migrants or denigrated workers.

On the other hand, the development of international migration flows has been explained by different theoretical models and different perspectives. The economic approach to migration emphasises the labour market and wage differentials between countries; world system theory analyses mobility as a product of globalisation; network theory considers that mobility is motivated by kinship and friendship ties; transnational space theory considers that migrants’ practices connect countries of origin and destination, and circulate ideas, symbols and culture (Mora, 2008; Portes et al., 2002). A novel approach is that of securitisation, which explores how states use the penal system to legitimise anti- immigrant policies, leading to increased irregular migration (Carrasco, 2024; Delkáder, 2022; González, 2021; Ortega, 2015).

### The securitarian turn in migration

Migration control policies have adopted the idea of security to manage migratory movements and hinder the settlement of migrant workers. These policies give States greater power to detain or expel migrants in an irregular administrative situation. The securitisation of international migration is a process that involves different actors, media, ministers, academics, journalists, experts (Carrasco, 2024; Delkáder, 2022). As well as the use of technology, increased military budgets and border control (Luiselli, 2016). With the discourse of protecting migrants and national security, governments justify programmes such as Frontex in Europe or the Southern Border in North America (Garcia, 2021). However, they have other effects: they allow for mass deportation, they present migration as a latent threat, they increase irregular migration, they turn irregular migration into criminal behaviour, and they generate new forms of punishment, surveillance and management of irregular migration in particular.

Waever (1989) explains that securitisation was restricted to military and inter-state threats. It followed a process whereby the press, or the government, presented the existence of supposed threats to the public in order to increase economic resources, armaments or police. This same process has been applied to migration. Ortega (2015) suggests that much of the public debate on irregular migration is biased, promoting the idea that migrants do not deserve social protection, nor do they have any rights at all. For example, when Frontex talks about vulnerability, it refers to borders, not to the people crossing them (González, 2021).

Although most empirical studies have shown that migrants tend to commit less crime, the US government has explicitly linked migrants to criminal activity. Trump has stated that “Norwegians are desirable, but Mexican ‘rapists’ are not” (Garcia, 2021, p. 113). Spain, for example, does not guarantee the rights and needs of irregular migrant children, and there is public and political acceptance that exclusion is justified by their irregular status (Ortega, 2015).

The 9/11 attack was the pretext that allowed the US to link irregular migration with security, terrorism and drug trafficking (Delkáder, 2022). However, Bigo (2002) explains that the securitisation of migration in the West began in the 1970s with the crisis and the integration of the European Union. Although Europe presumes to be humanist and opposed to physical walls, its practices are anti-immigration. shows how over the years Europe, with the aim of sealing its borders and discouraging irregular migration from outside the EU, has been reinforcing the Schengen borders and has created and financed the European Border System EUROSUR, Frontex and the Coast Guard. It also shows that the various controls follow ethnic, racial and class selection criteria.

The controls have not been carried out in terms of human needs, but rather for mass surveillance, to give greater freedom to the military power that acts against irregular immigration and, above all, to legitimise the state’s control of certain groups. The securitarian turn, therefore, allows us to analyse how, based on an anti-immigrant political climate, the state uses the legal system to control migratory flows. By linking the immigrant with criminal behaviour, it produces different markers: categories of desirable and undesirable immigrants; suspension of freedoms and rights for people who do not comply with migration laws; and symbolic denigration, under the label of “criminals,” “delinquents” and “rapists” (Garcia, 2021, p.115).

Once defined by the state itself, migrants become visible and can be managed, managed, degraded and overexploited in the labour market. The distinction of people by migration status is capitalised on by the labour market and politicians who, under nationalist, racist and xenophobic rhetoric, catalyse socio-economic problems into the image of the irregular migrant as a potential criminal.

Focusing on the political rhetoric that blames immigrants for altering the national order or putting the welfare system at risk, leads us to forget that crises and inequalities are generated by capitalism. A system whose distinctive features are its global expansion, accumulation, progressive and polarised development (Sandoval, 2018). Every time there is overproduction, crises appear. In order to reactivate accumulation, capitalism demands socio-economic, political and legal changes, which is why the state responds by using the penal system to manage crises and the surplus of the population.

The reinforcement of borders turns migration into a criminalised surplus dehumanised under the principle of racial classification of populations (Romero, 2018). This is important for the reproduction of capitalism, as it requires combining wage employment with different forms of labour, such as that performed by undocumented migrants, women’s reproductive labour or slave labour in workshops (Robinson, 2019).

According to Ambrosini (2015), society’s perception of the risks associated with irregular immigrants is selective. Immigrants may be perceived as dangerous to cities, but they are also tolerated for their usefulness and merits. In the case of domestic workers and caregivers, Ambrosini explains that they are tolerated because they work with native families, and because it is rare for them to be detained and deported.

Studies by Da Roit et al. (2013) and Da Roit et al. (2015) argue that the high demand for care work has encouraged migrant flows and irregular immigrants. Particularly in Southern Europe where care needs are covered by families. Countries such as Spain have poor or underdeveloped long-term care policies. This has led to a care market that employs mostly regular and irregular migrant women to take care of the care work not covered by the public system.

European policies consider that the employment of women is key to the sustainability of welfare states and the fight against poverty (Da Roit et al., 2013). The inclusion of women in the labor market is not always accompanied by a redistribution of domestic work. For example, the Spanish State reproduces the patriarchal logic, which attributes the burden of the production of services to women; and the racial logic, as immigrant women are the ones who cover the care work.

Studies such as those by Van Hooren (2012) argue that the Spanish state allocates relatively low public expenditure to care services, while private services remain expensive. Consequently, there has been an increased reliance on the migrant labor force that is relatively low-cost and often willing to work without formal employment contracts. These employment conditions result in differentiated categories of workers. Some enjoy full labor rights, while others possess few or none. The pandemic further highlighted how migration policies influence migrants’ access to social services and their vulnerability to labor exploitation.

### Talking to the women

The qualitative approach allows us to approximate the perceptions given by women about employment, health, food, or housing difficulties they faced during the pandemic. As well as the experience lived by women in their condition as immigrants and the access to administrative regularization. The empirical results we present here derive from qualitative research carried out in the context of the COVID-19 pandemic, in Barcelona.

The semi-structured interview was the main instrument of data collection. The design of the interview made it possible to gather information about the arrival of women in Spain, their administrative, labor, economic, and social situation. The interview was conducted in such a way that it was an account of the person, rather than answering questions from the researcher. The interviews lasted between 45 min and an hour. The coding of the interviews was carried out with the ATLAS-ti program. The codes that emerged from the interview were organized into eight categories: working conditions, experiences related to COVID-19, institutions, perceptions, housing, migration, types of employment, and survival strategies. Once the categorization was carried out, a conceptual map was created, which facilitated a more in-depth analysis of the empirical results. Although a large sample is needed to have a more general view of the experience of migrant women with the immigration laws, and the changes introduced by Law RD-L 16/2022, the testimonies of the informants show that not having a work and residence permit leads to inequalities.

In the city of Barcelona, there is a large contingent of migrant women, in regular and irregular administrative conditions, and with a long history in precarious jobs. With the aim of knowing the impact of the pandemic on women with different projects and trajectories, we looked for groups of women of immigrant origin. However, the pandemic made it difficult due to the lack of access to people willing to participate in the study. Finally, the eight women workers were contacted through the snowball technique and in contact with entities.

The interviews were conducted between March and June 2021, and in the context of the pandemic. Five of the interviewees were first-generation immigrants in Barcelona, aged between 27 and 55, from Latin America and one from Morocco. One of the participants of immigrant origin, Rosa, was also a spokesperson for the Collective of Women care worker, a collective that provides support in labour, housing, administrative and care issues. Laura is a member of the soup kitchen, which is made up of volunteers who work in the kitchen and accompany the users. Clara is the spokesperson for the Recycling Network, a network of neighbourhood help, located in a neighbourhood in Barcelona with a high percentage of immigrant population. The network recycles and distributes food, but it grew during the pandemic because of the needs of families in the neighbourhood who were left unemployed, or because informal jobs did not allow them to survive during the state of emergency.

The five participants from Latin America have in common that they entered Spain regularly and then became irregular, except for Mercedes, who arrived from the Dominican Republic regrouped by her partner. Isabela, María and Fernanda have Spanish passports. They managed to regularise their administrative situation at the beginning of 2000, before the current Law on Foreigners came into force, which began to require employment contracts or proof of monthly income (Gil Araujo, 2009). The other women have been regularising their status through social roots and after working without a contract, with the exception of Fatima who migrated from Morocco with her partner and two children. At the time of the interview, Fatima had been working for two years and had not been able to regularise her status.

Before emigrating, four of the participants were university graduates, the others had reached secondary school and high school. All of the women interviewed have had employment trajectories marked by the precariousness of their work, as a result of the laws on foreigners. It should be noted that all personal data, as well as other identifiable data, have been coded in order to preserve the confidentiality of the individuals.

### Labour trajectories marked by the legal act

Research by Pedone et al. (2012), Riopedre (2016), and Salas (2019) explains that the massive entry of Spanish women into the labor market has not been accompanied by adequate growth in public care services, nor by an equitable distribution of domestic and care work. Spanish families tend to hire women of different origins, socio- economic status and administrative situation to care for the elderly, children, for cleaning, feeding or companionship. This reproduces a class, racial and gender order in the care and services market.

The commodification of care led to changes in immigration policies. For example, Organic Law 4/2000, of January 11, 2000, on the rights and freedoms of foreigners in Spain and their social integration (BOE-A-2000-544), allowed the transfer of immigrant women to Spain to provide care services and was practically the only way to enter the labor market and regularize jobs. It should be noted that after the pandemic this Law was revised, and modified, with the aim of improving the working conditions of people employed in the care sector, by equating it with the common regime that gives access to social security (Molina, 2025).

At the time of conducting the interviews, the legal system in force was Organic Law 4/2000, of January 11, which presents deficiencies. The women interviewed with formal contracts were within a labor regime that did not provide access to unemployment insurance, allowed dismissal without just cause, without compensation, and precarious wages (Díaz, 2016). As one of the participants explained.

“While public policies do not demand that there be housework and care work with decent wages, people hire people to exploit them. As I was telling you, it does not pay what it should be, it pays what it can pay” (Fernanda, 21 years old in Spain).

In the trajectories of the women interviewed, we found that the first job with which they started their working life was home care work. In their accounts, the women agree that they accept a job that lacks fair working conditions and salaries, because it is one of the few legal loopholes that allows access to regularisation. These accounts coincide with Ambrosini (2015) research, who explains that, for newly arrived migrants, regularly or irregularly, the care economy makes it possible to cover housing needs, income, and the possibility of regularization.

The law on foreigners, officially Organic Law 4/2020, is the framework that regulates immigration in the Spanish state and grants the status of “regular or irregular immigrant,” “resident,” “refugee.” These categories define access to legal residence and basic services (Contreras, 2016).

This Organic Law includes the legal figure of *arraigo* (a form of residency based on social, family, or labor ties), which provides residence permits to individuals in an irregular administrative situation. In order for *arraigo* to be granted, there must be evidence of a social, economic, or family connection between the immigrant and Spanish society. For economic migrants, obtaining a labor contract is therefore a key requirement. It is worth noting that, following the COVID-19 pandemic, the Immigration Law underwent significant reforms aimed at improving access to residency. Royal Decree 1155/2024, of November 19, eliminated the previous requirement of a one-year employment contract. Currently, contracts with a duration as short as one month are accepted.

With the new criteria, it is expected that many individuals will benefit and gain access to residency through employment ties; however, in practice, this remains challenging. In sectors such as the care economy or cleaning, formal hiring is rare. Despite the reduced duration required for contracts, it remains difficult to find employers willing to engage in formal hiring practices. Lacking a formal employment contract creates serious obstacles, such as difficulties in finding housing.

The women interviewed explained that, in Barcelona, the minimum requirements set by real estate agencies to rent a house are as follows: a permanent employment contract is necessary; if the job is temporary, a guarantor is required; and a minimum income of €1,800 per month. Considering that the minimum interprofessional salary in 2021 was €965 per month, not everyone can afford housing. Moreover, even if immigrant women have the financial means, they rarely find a native guarantor.

“Of course he earned a lot of money, but in the black. I didn't have any collateral or contracts either. […] I've been here for 5 years, but the owner told me that they were never going to rent it to me… I had 4 jobs, and he told me “Yes, but I can't rent it to you, but if you give me a guarantee I'll rent it to you” (Isabela, 14 years old in Spain).

Immigrants often find it easier to rent a room in a shared household than to secure an entire apartment. However, what proves difficult to obtain is the *empadronamiento*—that is, the official registration of residence in Spain. The *empadronamiento* is a key requirement for accessing social *arraigo*, which in turn leads to administrative regularization. The interviewees explain that there are tenants who *charge 100, 200 euros for registration* (Rosa, migrant women’s collective). Housing also allows access to family reunification. The migrant women explain that it is difficult to meet the regularisation requirements demanded by the immigration authorities, such as having a job, getting a contract, three years for social roots, and that employers are willing to support them so that they do not remain in an irregular situation.

The participants indicated that once they had regularised their migration status, they sought to change sectors, some succeeded, others were able to do a series of precarious formal or informal jobs, and others were left at the mercy of their employers. Often women are forced to accept the conditions imposed either because of the promise of.

getting the contract, because it is the only source of livelihood, or because they depend on the contract to maintain their regular migrant status.

“The leave was for 15 days, and they called me on the third day. First, they called me to see how I am, and then they put pressure on you, telling me that the company is not an NGO. I tell them, it's not that I don't want to go to work, it's that I can't. My hands hurt. When they saw that I could not go to work, they told me that the company which is in the town would go. And I said: “Oh! They are going to fire me! I won't be able to renew” (María, 22 years old in Spain).

Caring, cleaning houses and hotels are jobs that require physical and mental effort. This daily strain takes its toll on women’s bodies. Interviewees reported some episode of job- related physical injuries such as carpal tunnel syndrome, tendon fractures, shoulder or tendonitis. Several authors (Bover et al., 2015; López and Ferrandez, 2021) argue that these illnesses tend to appear throughout the working lives of women employed in these sectors. In Isabel’s experience, the disease became an economic problem, as she worked informally, and only when she managed to save for the recovery period did she undergo the operation.

“My hands were damaged, I got carpal tunnel, which usually happens to those of us who clean, this happens to us. But I had to program myself to be able to go 4 months without receiving income because I could not work. So, I saved money because I could not give myself off work either, I did not have a contract” (Isabela, 14 years old in Spain).

Another element that appears in all the interview narratives is the deterioration in mental health. Work overload, lack of stability and low-income lead to pressure, stress, anxiety and depression. During the interviews in the care sector, it was common for employers to dismiss employees without prior notice. For example, Fernanda (21 years old in Spain), cared for a child, but was dismissed after three months because the family claimed that the child had developed a strong bond with her. Maria (22 years old in Spain) recalls that her first job lasted 3 months, the family replaced her with someone else.

### Administrative status: a mechanism that makes life more precarious

We have pointed out that a distinctive feature of capitalism is that free wage labour with rights covers few populations in the world, because it requires other forms of work (Robinson, 2019). Among non-EU nationals living in Spain, regularity and stability in their administrative situation is a constant quest. But the figure of employment roots is key to maintaining inequalities and precariousness. It is an inequality that comes from the state itself, whose constitution is based on the classification between the European Community subject and the non-EU subject. Differentiated bodies are constructed, so that they carry out different occupations. In the case of immigrants, they work in precarious, undervalued and poorly paid jobs.

“I started working very young. As time goes by, you don't feel anything [muscle pain]. Look how you end up now. Do you think they value you? Since I got sick, none of my bosses have called me. And the insurance company did not want to give me sick leave as a work accident. The insurance company wanted to send me to work with a bad arm” (Fernanda, 21 years old in Spain).

Díaz (2016) research explains that the economic crisis that began in 2008 and ended in 2014 will make it more difficult for care workers to maintain and apply the changes established in the 2011 law on foreigners. The law obliges people to have a written contract with a salary that is not below the minimum wage. This requirement, in the context of the pandemic, was difficult to meet.

During the pandemic, individuals with regular immigration status faced the risk of reverting to irregular status. For example, Fatima and Mercedes lost their residency status during the pandemic crisis because they were unable to get a new employment contract. Mercedes explains that she fell into irregularity when her partner, who had reunited her, became unemployed and she was fired from her job. During her confinement, Mercedes had job opportunities, but she was not hired because she did not have the proper documentation. As a result, they fell into food insecurity and residential insecurity.

Paperwork was a differentiating factor during the pandemic. Residence permits and formal employment gave access to social guarantees, such as the social benefits that three of the participants received: one for sick leave, one for ERTE (Temporary Labor Force Adjustment Program), and one for unemployment. Isabela reports that in 2020 she covered a substitution in a supermarket with a general scheme contract, which allowed her to continue paying for housing as she became unemployed for the first time in 14 years.

“With the pandemic, fortunately, I was able to apply for unemployment insurance, looking how valuable a general regime contract is. They gave me 6 months of insurance. I started looking for houses to clean, nobody wanted me to go home because of the pandemic” (Isabela, 14 years old in Spain).

On the other hand, the lack of social guarantees, due to the irregular administrative situation, accentuates the precarious living conditions. For Fatima, the pandemic has added to the already existing difficulties such as poor knowledge of the language, the short time she has been in Spain and her irregular administrative situation. Fatima, Isabela and Mercedes found it difficult to re-enter the labour market. During their confinement, hotels and restaurants remained closed, and the care and service economy did not offer work either. This gave rise to concern and uncertainty about the future.

“I don't work [laughs], with the cóvid-19 it has been very complicated to look for work. There was no work, there were no restaurants, (…). Restaurants were closed, hotels were closed, maybe if they weren't there I could find work as a cleaner in hotels, but since everything is closed, even if you have papers. It affected a lot” (Fatima, unemployed, 2 years old in Spain).

### Strategies developed by women in times of pandemics

Social crises show us how institutions, charged with implementing social policies, are subject to a protocolisation and bureaucratisation that has a negative impact on the people who seek these services. In times of crisis, institutions do not act sufficiently as material and emotional support for people with a high degree of vulnerability. For this reason, self-management and community networks become sources of support for the demands of women, collectives or neighbourhoods.

Moreno-Colom and López-Roldán (2018) argue that, although in times of crisis the number of unemployed migrant women increases, they also suffer less unemployment than immigrant men. Precisely because the commodification of care makes domestic work an indispensable activity. Those women who did not lose their jobs were subjected to relations of control and abuse in the workplace. This was one of the reasons why women’s emotional support became particularly relevant in the pandemic.

“We have experienced loneliness, we have experienced the violation of rights here, being exposed to a lot of situations. So, if we can create alternatives, we make them” (Rosa, collective spokesperson for the migrant women's collective).

The women interviewed explained their experiences of stress related to health, employment or unemployment, the economy, food, rent and household expenses. Rosa, spokesperson for the migrant women’s collective, explains that the women most affected emotionally have been the domestic workers, because they have experienced the pandemic alone. The migrant women’s collective activated instant messaging applications, with the aim of accompanying and coping with the fear of dismissal, homelessness, long working hours without rest, and exposure to greater types of abuse.

“We aim to accompany and support those who are left on the streets because they are fired from their jobs, or because their husbands die from one day to the next, or because they are in a very critical situation. So, they need to stop for a while to recover their territory and their bodies” (Rosa, collective spokesperson for the migrant women's collective).

Solidarity and bonds, two elements that women have repeated as a strategy to cope with different crises. The informants consider that all people can be excluded from policies or institutions. The women’s proposal is a political change from below. They are aware that social policies do not solve several problems that are systemic, and that they do not guarantee access to basic social services, such as energy rights or access to decent housing. For example, Isabel explains that she was unable to pay all her housing expenses. This led her to incur a debt of energy expenses since the beginning of the pandemic.

“They can't cut off my electricity, as I am at risk of exclusion. But they won't forgive the debt. I asked, “What will happen to the debt? “Don't worry, the debt will not be forgiven”, but they will give you up to 2 years to pay. This still makes me a bit overwhelmed” (Isabela, 14 years old in Spain).

Participation in social networks and interpersonal bonds allows women to face living and working conditions (Ambrosini, 2015). The migrant women’s collective, together with other community networks, created resistance boxes aimed at cushioning the economic impact on women with higher levels of vulnerability, or those requesting support. In addition, strategies related to food emerged. For example, the Neighbourhood Food Network was created during the pandemic, as a response to the difficulties and food needs experienced by residents of a neighbourhood with a mainly immigrant population.

The Food Network operates in a self-managed way, and understands food as a right, based on politicisation and horizontality. This network appears as an alternative to institutionalised food practices, such as the cash card or the food bank, which operate bureaucratically. Whereas the food network does not demand any requirements, accreditation or identity documents from the people who attend. What the network seeks is to achieve horizontality in its practices.

“We are in a moment of restructuring and re-engagement, to be able to move from a welfare model to a network model where everybody is collaborating. We don't have to force everyone, but we have to make it more horizontal. That's the idea, to put it into practice, although it obviously has its problems” (Laura, Food Network spokesperson).

We note that both the Migrant Women’s Community Network and the Food Network, in their alternative practices, aim to bring about a transformation in the situation of the people (Llobet et al., 2020), and the agency of the users, as Mercedes explains.

“I come to [Food Network] as a volunteer, they give me food, fruit, vegetables… And so, with my 400 euros [from the subsidy] […] Sometimes, I do not recycle and I come, and they give me my food. Because I earn it and because I need it, of course” (Mercedes, 5 years old in Spain).

Outside the community networks, other strategies mentioned by the women were the search for financial aid, negotiation to reduce the price of rent in a pandemic situation, sharing a flat and training for employment. For example, one of the interviewees, when she became unemployed due to the pandemic, started occupational training courses and Catalan studies, with the aim of change the care work and getting a better salary income.

### Concluding remarks

The empirical results analysis in this paper, reveal that the vulnerability faced by migrant women during the pandemic is the result of pre-crisis conditions and is directly related to the Law on Foreigners. In the context of Covid-19, although it is true that women with regular administrative status had access to certain social benefits. However, in some cases they were insufficient to survive the coronavirus crisis. Basically because, with the crisis, the labour market was practically at a standstill, and they were exposed to the risk of losing their residence permits.

On the other hand, the women interviewed who have suffered most from the impact of the pandemic have been those in an irregular situation. Women in the care sector have encountered different situations. Those who kept their jobs did so under the risk of infection; others were fired from fear that the worker would bring the virus into the household or because their families ran out of income. Particularly, women who became unemployed had problems paying or maintaining housing, fell into food and economic insecurity.

Stress was present in all the women, because of the fear of becoming unemployed, precarious, or irregular. The fear of not being able to renew the work and residence permit appeared in the women’s accounts. Let us recall that the residence and work permit is linked to a one-year work contract; if it is not held, there is no possibility of regularization. This has consequences for other areas of life, for example, being left without housing due to the lack of a work contract to prove income.

The pandemic exposed a greater precariousness for immigrant women without papers, without income, and depending on the resources that could be obtained from community networks. In these two cases, the community network was essential for the material and emotional subsistence of the women and their families.

Based on the experience of the interviewees, we observe that the policies that aim to cushion the effects of social exclusion, such as food aid with the wallet card, the extension of the energy payment or the certificate of risk of social exclusion, are insufficient. The precariousness of migrant women is due to the laws on foreigners, which becomes evident in the bureaucratised practices of the institutions, as they prevent people in an irregular situation from accessing this aid, because they demand documents from those who often do not have them.

The strategies developed at the individual and collective level were fundamental for the survival and empowerment of the women. In the accounts of the interviewees, they emerged as vital spaces where they could share experiences, respond to housing or food needs where institutions do not reach. From a material, emotional and practical point of view, the community networks have been essential for the subsistence of migrant women and their families.

## Data Availability

The raw data supporting the conclusions of this article will be made available by the authors, without undue reservation.
